# Immunological memories of the bone marrow

**DOI:** 10.1111/imr.12656

**Published:** 2018-04-17

**Authors:** Hyun‐Dong Chang, Koji Tokoyoda, Andreas Radbruch

**Affiliations:** ^1^ Deutsches Rheuma‐Forschungszentrum Berlin a Leibniz Institute Berlin Germany; ^2^ Charité University Medicine Berlin Germany

**Keywords:** bone marrow, memory T cells, tissue‐resident memory

## Abstract

Memory for antigens once encountered is a hallmark of the immune system of vertebrates, providing us with an immunity adapted to pathogens of our environment. Despite its fundamental relevance, the cells and genes representing immunological memory are still poorly understood. Here we discuss the concept of a circulating, proliferating, and ubiquitous population of effector lymphocytes vs concepts of resting and dormant populations of dedicated memory lymphocytes, distinct from effector lymphocytes and residing in defined tissues, particularly in barrier tissues and in the bone marrow. The lifestyle of memory plasma cells of the bone marrow may serve as a paradigm, showing that persistence of memory lymphocytes is not defined by intrinsic “half‐lives”, but rather conditional on distinct survival signals provided by dedicated niches. These niches are organized by individual mesenchymal stromal cells. They define the capacity of immunological memory and regulate its homeostasis.

## 1 MEMORY PLASMA CELLS

From a personal point of view, we became interested in the lifestyle of memory lymphocytes when we started to analyze the persistence of plasma cells in the bone marrow.^1^, ^2^ Plasma cells had been identified as antibody‐secreting cells in 1947.^3^ It had been noted early on that they disappear from secondary lymphoid organs when immune reactions are finished,^3^, ^4^ an observation leading Astrid Fagraeus to state that the plasma cell is “a cell which has already passed its greatest functional activity”.^3^ A second observation apparently supported this view, in that plasma cells isolated from secondary lymphoid organs died rapidly in cell culture, unlike other lymphocytes.^5^, ^6^, ^7^, ^8^ The apparently short lifespan of plasma cells created a conceptual problem, namely how to explain the persistence of specific antibody titers in the blood, long after termination of an immune response.^9^ One solution to this problem would be if immune reactions would not terminate, but continue to smolder, driven by residual antigen, at very low to undetectable concentrations.^10^ Plasma cells would have a defined short half‐life and dying plasma cells would be replaced constantly and over time, by newly generated ones. In other words, persistent humoral antibody titers would not reflect “memory” but rather a chronic immune reaction. In a modification of this concept, Lanzavecchia and colleagues later postulated that bystander activation of memory B lymphocytes could generate plasmablasts replenishing the ranks of dying plasma cells.^11^ In summary, the prevailing concepts say that plasma cells have a defined, short half‐life, and that in order to maintain persistent antibody titers, ie, “humoral memory”, their numbers would have to be replenished constantly, either by cognate or bystander activation of B lymphocytes, their precursors.^12^

An alternative concept had been suggested in 1974 by Benner and colleagues, who had observed that, while plasma cells disappeared from secondary lymphoid organs when an immune reaction to a defined antigen terminated, plasma cells specific for that antigen could easily be detected in the bone marrow at later time points.^13^ Twenty years later we showed that plasma cells generated in a defined murine immune reaction were thereafter maintained in the bone marrow for up to 120 days, as cells resting in terms of proliferation, in constant numbers, reflecting about 10%‐20% of the originally generated plasma cell population.^1^, ^2^, ^14^ At about the same time, Slifka and colleagues, using an entirely different technical approach, showed that plasma cells of a given immune reaction would persist in murine bone marrow, even if their regeneration from activated B lymphocytes was blocked.^15^ Similarly, treatment of human patients with Rituximab did not ablate humoral memory. Rituximab is an antibody depleting circulating B lymphocytes which express CD20, but not plasma cells which do not express CD20. Titers of serum antibodies to measles or tetanus remained constant.^16^ Also in mice, depletion of CD20‐expressing B lymphocytes affected neither the numbers of bone marrow plasma cells nor the persistence of antibody titers.^17^ Recently, assessment of lifetimes of individual plasma cells in the bone marrow of macaques has confirmed that those plasma cells can persist there for a decade.^18^ Similarly, they can survive for decades in the human gut,^19^ and it has been discussed that long‐lived mucosal plasma cells may play a role in maintaining the mucosal microbiota.^20^ Apparently, plasma cells can also persist in inflamed tissue, as in the inflamed kidneys of NZB/W mice^21^, ^22^ or the inflamed central nervous system of mice with experimental autoimmune encephalomyelitis.^23^ During acute inflammation in the inflamed tissue, the persistence of plasma cells, in particular those secreting antibodies specific for pathogens driving the inflammation, would provide local protection. In chronically inflamed tissues long‐lived plasma cells secreting autoantibodies might themselves drive the inflammation. Pathogenic long‐lived plasma cells are a novel therapeutic target.^24^, ^25^

The persistence of long‐lived plasma cells over long time periods qualifies them as true “memory” cells, memorizing the original antigenic challenge independent of continued antigenic stimulation,^1^ ie, maintaining information in the absence of the original instruction. We therefore suggest to designate them “memory plasma cells”, replacing the operational term “long‐lived” plasma cells used so far in the literature. Whether or not the precursors of these memory plasma cells need an intrinsic “competence”, other than the capacity to find the path towards a memory niche, remains to be shown. So far, it appears that most if not all memory plasma cells are derived from memory B lymphocytes, either in extended primary or in secondary immune responses, and that their generation is dependent on help from T lymphocytes (reviewed in Ref.^26^).

Memory plasma cells are diverse regarding expression of different classes of antibodies, different chemokine receptors and other surface receptors.^27^ While their precursors express CD38 at rather low levels, memory plasma cells express high levels of CD38.^28^, ^29^ This CD38 is apparently distinct from CD38 on other cells; it is selectively recognized by a plasma cell‐specific antigen receptor of lamprey, because it is dimerized and shows NAD glycohydrolase activity.^30^ Within CD38^high^ plasma cells of the bone marrow, further diversity has been described regarding expression of other surface molecules.^28^ Among those CD19 has raised some interest recently as a candidate marker for memory plasma cells maintaining long‐term memories,^31^, ^32^ similar to memory CD4^+^ T lymphocytes of the bone marrow, as will be discussed later.^33^ Apparently loss of CD19 expression can occur already at the transition from plasmablast to plasma cell^34^. Whether or not CD19 qualifies as a target for the selective ablation of memory plasma cells secreting pathogenic antibodies remains to be shown.^35^

## 2 CONDITIONAL SURVIVAL OF MEMORY PLASMA CELLS—THE MEMORY NICHE

Analyzing the lifestyle of memory plasma cells in more detail immediately challenged a second traditional viewpoint, namely the concept of an intrinsic “half‐life” of memory lymphocytes. Isolated plasma cells of murine bone marrow survive only for a day or 2 in culture medium, unless this medium is supplemented with cytokines and stimuli for adhesion receptors, like CD44.^7^ In vivo, these plasma cells can essentially survive for a lifetime, as discussed above. Clearly, bone marrow plasma cells have no intrinsic “half‐life”, but rather survive depending on signals from their environment, most likely cytokines and ligands for adhesion receptors.

The “survival code” of memory plasma cells is still poorly understood. An essential signal is activation of the B‐cell maturation antigen (BCMA/CD269) by one of its ligands, B‐cell activating factor (BAFF/BLyS/CD257), or a proliferation inducing ligand (APRIL/CD256).^36^ BCMA is encoded by the tumor necrosis factor receptor superfamily member 17 gene (*TNFRSF17*), and signaling through the NF‐κB pathway regulates expression of the myeloid leukemia cell differentiation protein MCL1, which is essential for survival of bone marrow plasma cells.^37^ A second essential signal seems to be integrin α4β1 (VLA‐4), a dimer of CD49d (α4 integrin) and CD29 (β1 integrin) and receptor for vascular cell adhesion molecule 1 (VCAM‐1/CD106) and fibronectin, and the lymphocyte function‐associated antigen 1 (LFA‐1), a dimer of integrin αL (CD11a) and integrin β2 (CD18) and receptor for intercellular adhesion molecule 1 (ICAM‐1/CD54). Co‐administration of antibodies to LFA‐1 and VLA‐4 in mice led to a depletion of bone marrow plasma cells.^17^ It remains enigmatic which of the 2 adhesion molecules, VLA‐4 or LFA‐1 of plasma cells, is providing the essential survival signal, and how it is acting in the plasma cells to prevent apoptosis, or whether both are redundant. It should be noted that inhibition of CD49d alone, by the therapeutic antibody Natalizumab, neither affected the generation nor the maintenance of humoral memory in secondary immune reactions to vaccines in patients with systemic sclerosis.^38^ Neither did it decrease Aquaporin‐4‐specific autoantibody titers in patients with neuromyelitis optica spectrum disorders. Those autoantibodies were presumably derived from established memory plasma cells, at least in those patients who before or afterwards were refractory to Rituximab.^39^ It would not be too surprising if adhesion between plasma cells and stromal cells was mediated by redundant receptors. Likewise, as discussed above, BCMA can be activated by either April or BAFF.^36^ VCAM‐1 and ICAM‐1, the ligands of VLA‐4 and LFA‐1, are expressed by bone marrow stromal cells^40^ and, expectedly, in the bone marrow most if not all plasma cells contact stromal cells expressing VCAM‐1.^41^, ^42^ Mesenchymal stromal cells thus qualify as organizers of a survival niche for plasma cells, probably by providing essential survival signals, namely integrin‐mediated cell contact. Integrins are known to signal via the phosphatidylinositol‐3‐kinase (PI3K)/protein kinase B (AKT) pathway,^43^ and initial evidence has been reported that this pathway indeed may support plasma cell survival.^44^ However, the mTOR pathway, one of the targets of PI3K/AKT signaling, does not seem to be required for the persistence of memory plasma cells.^45^

The dialogue between mesenchymal stromal cells and memory plasma cells of the bone marrow is probably much more sophisticated, with at least 2 additional aspects that are essential for understanding the organization of humoral memory. First, it is obvious from histological inspection that in the bone marrow, plasma cells are not clustered but dispersed individually throughout the parenchyme.^42^ Apparently 1 stromal cell cannot host more than 1 memory plasma cell. The molecular basis for this restriction is probably independent of integrins and completely enigmatic, but the consequences are not. This restriction means that stromal cells limit the maximum number of memory plasma cells. Less than 5% of the bone marrow cells are stromal cells, their numbers relate to the volume of bone marrow and blood and to the size of the individual vertebrate. This simple and efficient regulatory mechanism provides a ceiling for the serum concentrations of antibodies of humoral memory, by counting and limiting the numbers of memory plasma cells. It is also obvious that once the ceiling is reached, newly generated plasmablasts have to compete with established memory plasma cells for habitation of niches. While direct evidence for such competition is missing, indirect evidence comes from the mobilization of “old” plasma cells of diverse specificities into the blood during intentional vaccinations of humans.^46^

A second aspect of central relevance is the apparent immobility of memory plasma cells. Plasmablasts within the first week after their generation are highly mobile and migrate toward gradients of chemokines addressing their chemokine receptors. Of particular relevance for their attraction to the bone marrow within this timeframe is the chemokine CXCL12, a ligand for the chemokine receptor CXCR4 of plasma cells. CXCL12 is expressed by those mesenchymal stromal cells which the plasma cells contact in the bone marrow.^41^ The CXCR4 antagonist AMD3100 blocks the immigration of plasmablasts into and the establishment of memory plasma cells in the bone marrow, strongly suggesting that the plasma cells had been attracted to the stromal cells by CXCL12^47^. In humans (re)vaccinated against tetanus, a wave of newly generated plasmablasts is detectable between days 6 and 8 after vaccination in the blood. In transwell migration assays, these plasmablasts migrate toward CXCL12 gradients.^46^ Outside of this time window, only few plasmablasts and plasma cells are detectable in blood, and most if not all of them are obviously derived from mucosal immune responses.^48^ In mice, as early as 2 weeks after their generation, plasma cells have lost their mobility, they do not move within the bone marrow,^42^ and when isolated from the bone marrow, they do not migrate toward gradients of CXCL12 in transwell migration assays.^14^ This is even more surprising, since they still express CXCR4, and react to CXCL12 by improved survival in tissue culture.^7^ The immobility of memory plasma cells implies that once their survival niche disintegrates, memory plasma cells will die, because they are not able to move to alternative niches. In regenerating tissue of acute inflammation, this may help to get rid of tissue‐resident plasma cells of the acute immune reaction, ie, terminate the peak response. It also may contribute to the age‐dependent decay of humoral memory.^49^, ^50^

Although stromal cells are a key element of the memory plasma cell niche, they are not the only element. Stromal cells do not express BAFF or APRIL themselves, and it is obvious that those ligands of BCMA, the second essential survival signal for memory plasma cells, have to be provided by other cells, which we have termed “accessory” cells of the niche. Eosinophilic granulocytes,^51^ basophilic granulocytes,^52^ megakaryocytes,^53^ and monocytic cells^54^ have been described as accessory niche cells. Priorities and redundancies among these cell types have not finally been resolved.^55^, ^56^ It should be noted that even in sublethally irradiated mice, ie, in bone marrow with a significant ablation of hematopoietic cells, memory plasma cells can survive several days until the hematopoietic cells have been regenerated again.^42^

## 3 CIRCULATING MEMORY T LYMPHOCYTES

At first glance, the lifestyle of memory plasma cells, their persistence as resting and resident cells in niches of the bone marrow, with no intrinsic half‐life but rather surviving dependent on distinct signals, differs completely from that of other memory lymphocytes. In contrast to memory plasma cells, antigen‐experienced T lymphocytes are easily detectable in secondary lymphoid organs and in the blood. During an immune reaction, their numbers rapidly expand. When the reaction is over, their numbers contract again, first rapidly, then slowly.^57^, ^58^, ^59^ The “half‐life” of blood‐borne antigen‐experienced T cells in the phase of slow contraction shows a tremendous variation, ranging from 8 to 15 years, upon smallpox vaccination of humans^60^ to less than 40‐60 days, in mice immunized with a peptide of lymphocytic choriomeningitis virus or ovalbumin.^59^ In mice, the numbers of adoptively transferred and experienced CD4^+^ and CD8^+^ T cells decline with half‐lives of 15‐70 days, in the absence of antigen.^61^, ^62^ Such adoptively transferred CD8^+^ or CD4^+^ T cells can provide protection.^61^, ^63^, ^64^, ^65^, ^66^

The classification of antigen‐experienced vs circulating memory T cells is still on shaky ground. For human T cells, the classification is according to expression of CD45 isoforms, with memory T cells expressing CD45RO rather than CD45RA.^67^, ^68^ In mice, memory T cells have been classified according to low expression of CD45RB,^69^, ^70^ and stable acquisition of high CD44 expression.^71^ Taking a closer look at the functional potential of circulating antigen‐experienced T lymphocytes, Sallusto and Lanzavecchia classified them as “central” memory cells, expressing the chemokine receptor CCR7, and “effector” memory cells not expressing CCR7.^72^ CCR7 is involved in the organization of immune reactions in secondary lymphoid organs.^73^ Thus, central memory T cells would provide a memory for secondary immune reactions, while effector memory T cells would provide immunity directly. In the meantime, the zoo of circulating memory T‐cell types, classified according to phenotype and (presumptive) function, has expanded.^74^ It includes CD45RA memory stem cells^75^, ^76^ and CD45RA “terminally differentiated” effector memory T cells.^77^, ^78^ This zoo may even expand further in the future, considering that among naive T lymphocytes, those expressing CD31 are truly naive, ie, recent thymic emigrants, while those not expressing CD31 have lost their T‐cell receptor recombination excision circles.^79^, ^80^ This indicates that they had been activated to proliferate in the periphery. It remains to be shown whether this activation was triggered by (auto)antigen or not.

In terms of global gene expression, memory T lymphocytes of blood are resting,^81^ and clearly distinct from reactivated memory T cells. Activation by antigen, however, leaves a lasting impression on antigen‐experienced T cells. Depending on the kind of the original and subsequent activations, they become epigenetically imprinted to express particular genes in future restimulations. Imprinting occurs on at least 4 levels: the induction of transcription factors, the induction of regulatory RNA, the modification of chromatin, and the demethylation of DNA. Ever since the original classification of circulating memory CD4 T cells into Th1 and Th2 cells,^82^ functional compartmentalization of antigen‐experienced T cells has become a favorite exercise of immunologists.^83^, ^84^, ^85^ We have shown that GATA3 mediates the epigenetic imprinting of the interleukin‐4 (*Il4*) gene,^86^, ^87^ and how imprinting of the interferon‐γ (*Ifng*) gene is induced by STAT4.^88^, ^89^ Most importantly, we and others have shown that imprinting of cytokine genes in activated naive T cells but not in memory T cells requires progression through the S phase of the first cell division. This is suggesting that imprinting is indeed linked to epigenetic modification of DNA and would explain why cytokine gene expression is faster in secondary than in primary immune reactions.^90^ Essentially everything that we think we know about memory T lymphocytes has been analyzed on T cells obtained from murine secondary lymphoid organs, mostly spleen, or human blood. These cells were and still are considered by many as representatives of memory T lymphocyte populations circulating through the body in quest for cognate interactions, looking for cells presenting them their antigen. This is surprising as already in 1964, McGregor and Gowans demonstrated that secondary immune responses are not impaired when all circulating lymphocytes are removed by chronic thoracic duct drainage.^91^ Yet, considering that circulation is their lifestyle, usually cells from spleen are adoptively transferred into blood, to analyze their persistence again in the spleen of the host.^61^, ^63^

It therefore comes as no surprise that the existence of professional memory T cells as such has been questioned, as compared to an expanded, slowly contracting population of circulating, resting effector T cells.^92^, ^93^

## 4 THE LIFESTYLE OF CIRCULATING MEMORY T LYMPHOCYTES

The lifestyle of circulating antigen‐experienced T lymphocytes is far from clear. Retention times in the blood and in the tissues are not known, nor is it known which tissues they attend, in which order, what keeps them there or releases them again? The prevailing concept of how circulating antigen‐experienced T lymphocytes are maintained is that of homeostatic proliferation, driven by cytokines, replacing memory T cells which die either due to a limited intrinsic half‐life, or by neglect, not being able to secure sufficient survival stimuli. The concept has been detailed out in a review by Surh and Sprent,^94^ summarizing evidence that interleukin‐15 is an essential cytokine for the maintenance of CD8^+^ memory T cells,^95^ while maintenance of CD4^+^ memory T cells is dependent more on IL‐7 than on IL‐15.^96^, ^97^ T‐cell receptor signaling seems not to be important for the maintenance of CD4^+^ memory T cells. CD4^+^ memory T cells persist when their TCR is ablated,^98^ and also in MHC class II‐deficient hosts.^99^ For CD8^+^ memory T cells, the situation is less clear. Their numbers decline upon ablation of their TCR.^98^ CD8^+^ memory T cells not expressing CD122 do, but CD122 expressing cells do not require MHC class I.^100^, ^101^, ^102^ Where the antigen‐experienced T cells of the circulating memory obtain the signals to proliferate, die, or exit a particular tissue is unclear. It has been discussed that the bone marrow may provide a hub for circulating memory T lymphocytes.^103^ IL‐7‐expressing cells are abundant also in the gut and in the lymphoid organs^104^ as are professional antigen‐presenting cells.^105^, ^106^

Persisting antigen, even at low doses, could contribute to the maintenance of antigen‐experienced T cells in a chronic immune reaction.^107^ Long‐term maintenance of antigen has been demonstrated for follicular dendritic cells^108^ and even in the absence of any detectable pathogen after viral challenge, antigen can apparently persist in amounts sufficient to stimulate adoptively transferred, naive T cells.^109^

On the other hand, antigen‐experienced T cells of murine spleen can survive and proliferate, when adoptively transferred into hosts which do not bear the antigen,^61^, ^63^ arguing that in the absence of antigen, circulating memory T cells are maintained and induced to proliferate by cytokines, ie, by homeostatic proliferation. Homeostatic proliferation of memory T cells from murine spleen has been analyzed by Becker and colleagues, who transferred splenic CD8^+^ T cells into naive hosts.^94^, ^95^, ^110^, ^111^, ^112^, ^113^ The transferred T cells homed to essentially every tissue analyzed, including the spleen, bone marrow, liver, and blood. Within 25 days, 30%‐60% of the cells in those tissues had divided at least once, according to loss of carboxyfluorescein succinimidyl ester label. This proliferation was dependent on IL‐15. We recently confirmed this finding, using cyclophosphamide to ablate proliferating CD8^+^ memory T cells in vivo. About 50% of all CD8^+^ memory T cells from spleen, as well as 50% of splenic memory CD8^+^ T cells generated in an intentional immune response, were ablated within 2 weeks of cyclophosphamide treatment.^114^ Interestingly, already 1 week of cyclophosphamide treatment was sufficient to ablate 50% of all CD8^+^ memory T cells from spleen, suggesting that 50% of the antigen‐experienced T cells of the spleen are rapidly proliferating, while the other 50% are not proliferating at all. Even more interestingly, this had not been indicated by the expression of Ki‐67, an antigen believed to be expressed by cells in the G1 to M phases of the cell cycle, but not by those resting in the G0 phase of the cell cycle.^115^ In summary, Becker and colleagues and our group, using adoptive transfer of labeled cells and in vivo ablation of proliferating cells, respectively, have provided compelling evidence that 50% of splenic antigen‐experienced T cells proliferate rapidly. Whether these are the circulating memory T cells or antigen‐experienced cells of recent or chronic immune reactions, and what discriminates them from the other 50% remains a challenge for future research. Are the 50% of memory T cells, which are not rapidly proliferating, a distinct population of resident and resting memory cells, or are they circulating memory cells “taking a nap” before going on another round of circulation? Recently the group of Rafi Ahmed could show by Deuterium labeling of human volunteers, that CD8^+^ memory T cells induced by yellow fever vaccination and circulating in the blood, are maintained as quiescent cells for more than a year.^116^

## 5 THE BONE MARROW—HUB FOR CIRCULATING OR HOME OF RESIDENT MEMORY T CELLS?

Another indication for homeostatic proliferation of splenic memory T cells had come from analyses of the uptake of bromodeoxyuridine (BrdU) into their DNA, taken as a surrogate marker for proliferation.^110^, ^111^, ^113^ In those experiments, about 50% of the splenic memory CD8^+^ T cells had incorporated BrdU within 14 days. However, these results should be interpreted cautiously. We could show that BrdU induces at least some of the proliferation it measures in antigen‐experienced CD8^+^ memory T cells.^117^ Feeding mice with BrdU resulted in a marked increase in Ki‐67^+^ CD8^+^ memory T cells, and cells in S/G2/M phases of cell cycle, according to staining of their DNA with propidium iodide. This was even more prominent for memory CD8^+^ T cells of bone marrow, a finding which led to an ongoing debate on the nature and lifestyle of memory T cells in the bone marrow.^103^, ^118^, ^119^, ^120^, ^121^, ^122^, ^123^, ^124^

Determining proliferation by other methods, bone marrow memory T cells are resting in terms of proliferation. According to staining of Ki‐67, more than 90% of murine bone marrow memory CD8^+^ T cells are in G0 of the cell cycle, more than 180 days after the onset of an intentional immune response, and less than 0.5% are in the S/G2/M phases of cell cycle.^117^ The same is true for human memory CD8^+^ and CD4^+^ T cells of bone marrow.^33^ And finally, ablation of proliferating memory T cells in mice, using cyclophosphamide, shows that within 14 days, memory CD8^+^ T cells of the bone marrow are not deleted at all, contrary to their splenic counterparts, as discussed above. This is true for memory T cells of an intentional immune response as well as for the entire population of all CD8^+^ memory T cells of the bone marrow, those generated by natural infections over time in these mice.^114^ It should be noted that this is true also in the presence of Fingolimod (FTY720), blocking sphingosine‐1‐phosphate‐mediated trafficking of lymphocytes, and emptying the murine blood of lymphocytes. These experiments identify most if not all CD8^+^ memory T cells of the bone marrow as resident cells, resting in terms of proliferation at least for the time of observation. Bone marrow is a quiet and privileged place, in that it is not connected to the lymphatic vessel system, and can be accessed and exited only via the blood stream.^125^ However, the presence of a minor population of circulating memory T cells cannot be excluded and it is likely that occasionally such cells pass by, as had been the case in the adoptive transfer experiments reported by the group of Rafi Ahmed.^110^

Evidence for a resident population in the bone marrow of memory CD4^+^ T lymphocytes is mainly based on their exclusive repertoire of T‐cell receptor specificities. In several intentional murine immune responses, antigen‐experienced CD4^+^ T lymphocytes relocated quantitatively to the bone marrow during the contraction phase, ie, within 60 days after onset of the immune reaction. After 120 days, antigen‐experienced CD4^+^ T lymphocytes were no longer detectable in spleen or lymph nodes, while in bone marrow, a stable population had been established.^59^ In contrast, the group of Jenkins described the preferential location of memory CD4^+^ T cells in the spleen and lymph nodes until day 160 after infection, in an immune response to *Listeria monocytogenes* infection.^126^ It should be noted, however, that from day 200 onwards, in that same figure, about equal numbers of antigen‐experienced CD4^+^ T cells were maintained in the bone marrow, as compared to the secondary lymphoid organs. Rather than pointing to “artificial” vs “real” memory, the different observations of Pepper and colleagues and Tokoyoda and colleagues point to a selective recruitment of antigen‐experienced CD4^+^ memory T cells to the bone marrow, dependent on yet poorly understood properties of the immune reaction.^127^

The selective recruitment to or survival in the bone marrow of memory T cells, reflecting “real” immunological memories, is even more obvious in humans. We compared frequencies and numbers of CD4^+^ memory T cells with specificity for distinct vaccines and infectious pathogens, in blood and bone marrow of the same individuals, by identifying antigen‐reactive T cells ex vivo.^33^ It turned out that in most adult human donors CD4^+^ memory T cells specific for viral pathogens encountered in childhood, either by infection or by vaccination, like measles, rubella, and mumps, were maintained exclusively in the bone marrow. Moreover, the very few cells detectable in blood showed a very limited scope of cytokine expression, while the cells of the bone marrow were polyfunctional, ie, they expressed several cytokines simultaneously. Memory CD4^+^ T cells recognizing a persistent virus, namely cytomegalovirus, were present both in blood and bone marrow, while memory CD4^+^ T cells recognizing pathogens of the skin, like Vaccinia and Candida, were more frequent in the blood than in the bone marrow. Such cells were presumably enriched in the skin,^128^, ^129^ although this has not been investigated in those donors. These differences in repertoire point to 1 potential sorting algorithm, namely archiving long‐term memories for systemic pathogens in the bone marrow, in the form of reactive, polyfunctional CD4^+^ memory T cells. The exclusive maintenance of memory CD4^+^ T cells specific for childhood vaccines/pathogens in the bone marrow also implies that those memory CD4^+^ T lymphocytes are not part of a pool of circulating memory CD4^+^ T cells, but rather permanent residents of the bone marrow.

## 6 THE LIFESTYLE OF BONE MARROW MEMORY T LYMPHOCYTES

The presence of antigen‐experienced T lymphocytes, both CD8^+^ and CD4^+^, in bone marrow has been known for quite some time. Such cells had been considered to be maintained by homeostatic proliferation or even cognate interactions with dendritic cells, as has been discussed before.^110^, ^113^, ^130^, ^131^, ^132^ Many of them express CD69 and some have upregulated expression of CD25. That is why they had been erroneously considered as proliferating cells in an “activated” state of memory.^133^

Recent evidence however suggests that resident memory T cells of the bone marrow are resting, not only in terms of proliferation (see above) but also in terms of activation. Their transcriptomes are those of resting cells.^33^, ^59^, ^81^, ^117^ CD8^+^ memory T cells of the bone marrow express only about 0.6 pg of RNA per cell, as compared to activated CD8^+^ T cells, which express more than 10 pg of RNA per cell.^117^ Genes encoding cytokines or cytolytic enzymes and those promoting proliferation are not expressed at detectable levels. Genes that had been described as “signature of tissue‐resident memory T lymphocytes”^134^ are expressed. Thus, at a global level of gene expression, memory T lymphocytes of the bone marrow are dormant, and distinct from circulating memory T cells. This is confirmed, when we look not at gene expression itself, but rather at epigenetic imprinting of genes for reexpression.^135^ This analysis reveals a progressive global demethylation for circulating central memory, effector memory, and terminally differentiated memory cells. Memory CD4^+^ T cells of the bone marrow are intermediate between circulating central memory and effector memory T cells.

If global gene expression indicates that bone marrow‐resident memory T cells are resting, what is the significance of expression of CD25 or CD69 by some of them? In humans, about 10% of memory CD4^+^ T cells circulating in the blood have upregulated expression of the α chain of the receptor for IL‐2 (CD25^high^). These cells also express the transcription factor forkhead box P3 (FOXP3) and have downregulated expression of the receptor α chain for IL‐7 (CD127). Taken together, this qualifies them as bona fide regulatory memory T cells.^136^ In bone marrow as well, about 10% of the memory CD4 T cells express CD25^high^, CD127^low^, and FOXP3,^33^ arguing that those cells are regulatory memory T cells.

CD69 is expressed in humans by about 30% of the CD4^+^ and 60% of the CD8^+^ memory T cells of the bone marrow.^33^ In mice, 30%‐40% of bone marrow CD8^+^ memory T cells express CD69,^117^ and about 40%‐50% of bone marrow CD4^+^ memory T cells.^137^ Expression of CD69 is induced upon activation of T cells^138^ and therefore has been conceived as a marker of activation, also for bone marrow memory T cells.^133^, ^139^ However, in murine bone marrow, neither CD69^+^ nor CD69^−^ memory CD4^+^ T cells are cycling according to Ki‐67 expression.^137^ In human bone marrow, both CD69^+^ and CD69^−^ memory T cells have transcriptomes of quiescent cells, with very few genes differentially expressed between them. Thus, both in terms of proliferation and activation, CD69^+^ memory T lymphocytes of bone marrow are resting.

One of the genes differentially expressed between CD69^+^ and CD69^−^ memory CD4^+^ T lymphocytes of bone marrow is the gene for the receptor for sphingosine‐1‐phosphate (*S1PR1*), which is not expressed by CD69^+^ memory T cells.^33^ CD69 is an antagonist of S1PR1, blocking the S1P‐mediated egress of lymphocytes from secondary lymphoid organs into the blood.^140^, ^141^, ^142^ It is remarkable that in CD69^+^ memory T cells of bone marrow, S1PR1 expression is blocked on the level of transcription, since it has also been reported that CD69 can directly block surface expression of S1PR1.^143^ In any case, CD69^+^ memory T cells of bone marrow are not equipped to sense sphingosine‐1‐phosphate, the chemokine attracting lymphocytes into the blood, and blood would be the only path to leave the bone marrow. They are “trapped” in the bone marrow. For CD69^−^ memory T cells of the bone marrow, it remains to be shown whether and if so, how they are maintained in the bone marrow.

Aside from its role in retaining memory T lymphocytes in the bone marrow, CD69 is essential for getting them into the bone marrow in the first place.^137^ CD69‐deficient CD4^+^ T cells, while mounting immune reactions comparable to their wildtype counterparts, failed to establish a population of bone marrow‐resident memory T cells. In the memory phase, memory T cells were absent from both spleen and bone marrow, and no efficient T‐cell memory was established. Adoptive transfer of CD4^+^ T cells activated in an intentional immune response, and deficient or sufficient for CD69, or stained for CD69, and thus blocking its interaction, revealed that CD69 is required for efficient translocation of activated T cells from the blood into the bone marrow. At present, the mechanism of this CD69‐mediated translocation is not clear. It remains to be shown whether recently described novel ligands of CD69, myosin light chains 9 and 12,^144^ are involved.

A second signal required for efficient translocation of activated CD4^+^ T cells is integrin α2 (VLA‐2, CD49b).^59^, ^145^, ^146^ CD4^+^ memory T cells stained for CD49b, and thus blocked for efficient use of VLA‐2, did not immigrate into the bone marrow in adoptive transfer experiments. VLA‐2 can bind to collagens I, II, and XI,^147^, ^148^ and collagen XI is exclusively expressed in the bone marrow. While on day 12 of an intentional murine immune response, CD4^+^ T lymphocytes in the bone marrow docked onto stromal cells expressing collagen II, on day 117, CD4^+^ memory T cells of that immune response were docking on to stromal cells expressing collagen XI, suggesting that VLA‐2 may be an essential adhesion molecule for the memory T cells to find a niche in the bone marrow.^145^ This observation also suggests that the lifestyle of resident memory T cells of the bone marrow might be not so different from the lifestyle of memory plasma cells. In murine bone marrow, memory CD4^+^ and CD8^+^ T lymphocytes are found in close association if not direct contact with stromal cells.^59^, ^117^, ^137^, ^145^ They are individually dispersed throughout the parenchyme.^117^ Average distances between any 2 CD4^+^ or 2 CD8^+^ memory T cells are the same as those between any 2 CD4^+^ and CD8^+^ memory T cells, suggesting that they compete for the same niches. And both, CD4^+^ and CD8^+^ memory T cells of the bone marrow are docking onto stromal cells expressing the adhesion molecule VCAM‐1 and the cytokine IL‐7.^59^, ^117^ Both, CD4^+^ and CD8^+^ memory T cells of bone marrow express VLA‐4, a receptor for VCAM‐1 and fibronectin. As it stands, the detailed molecular code of the bone marrow survival niches for memory T lymphocytes remains to be deciphered. In all likelihood it will turn out to be more diverse than anticipated at present. It also remains to be shown how the memory T‐cell survival niches of the bone marrow differ from the survival niches for memory plasma cells. We had originally anticipated that CXCL12‐expressing stromal cells provide niches for memory plasma cells, while IL‐7‐expressing stromal cells provide niches for memory T lymphocytes.^149^, ^150^ It may turn out that stromal cells organizing memory T lymphocytes do not only express IL‐7 but also CXCL12. The chemokine attracting precursors of memory T lymphocytes to the bone marrow has not yet been identified. It might be speculated that this is CXCL12, although no direct evidence has been published so far.

In summary, evidence is accumulating that defined populations of resident and resting CD4^+^ and CD8^+^ memory T lymphocytes of the bone marrow maintain immunological long‐term memories of systemic pathogens and vaccines. These memory cells persist in the bone marrow even when circulating memory T cells specific for those antigens have disappeared, showing that they are resident. Memory T lymphocytes of bone marrow are maintained in niches organized by stromal cells, similar to those niches maintaining memory plasma cells. Their precursors are antigen‐experienced T cells, immigrating from secondary lymphoid organs via the blood. For the precursors of CD4^+^ memory T cells, it has been shown that CD69 and VLA‐2 are essential for immigration and allocation to the niches. Whether all resident and resting memory T lymphocytes of the bone marrow continue to express CD69 or not is unclear at present. CD69‐expressing memory T lymphocytes of the bone do not express S1PR1, and thus are not attracted to the blood. Whether bone marrow is also traversed by circulating memory T lymphocytes, specific for those antigens recognized by memory T lymphocytes of the blood and the bone, remains to be shown. Parabiosis and adoptive transfer experiments would argue in favor of this, although both experimental approaches are not really modeling the steady state of immune memory.^123^, ^124^ For CD8^+^ memory T lymphocytes of bone marrow, however, it has been shown that they are not maintained by homeostatic proliferation, at the rates predicted by previous, paradigm‐setting work. Rather, by far the most if not all memory T lymphocytes of the bone marrow rest in terms of proliferation, like memory plasma cells, and in terms of transcriptional activity. Bone marrow‐resident memory T lymphocytes are the “Sleeping Beauty” of immunological long‐term memory to systemic antigens.

## 7 REACTIVATION OF BONE MARROW MEMORY T LYMPHOCYTES

When antigen‐experienced CD4^+^ T lymphocytes isolated from murine spleen or bone marrow were compared in adoptive transfer experiments for their competence to help B lymphocytes in a secondary immune reaction, bone marrow memory T cells were much more efficient than their splenic counterparts. While cells from the spleen did not provide help for affinity maturation of activated B lymphocytes, memory cells from the bone marrow did.^59^ At present this is the evidence that in secondary immune reactions, bone marrow‐resident memory T lymphocytes can play a decisive role.

The details of reactivation of bone marrow memory T cells are still largely obscure. In the memory phase, CD4^+^ memory T lymphocytes in their bone marrow niches are not located in the neighborhood of MHC class II‐expressing cells,^59^ precluding steady‐state activation via the TCR. Upon secondary immunization, CD4^+^ memory T lymphocytes specific for the antigen leave their niches and migrate toward MHC class II‐expressing cells.^151^ The reactivated, antigen‐specific memory T lymphocytes and the MHC class II‐expressing B lymphocytes and myeloid cells form clusters.^151^ The initial steps of this reactivation are not clear, but apparently they depend on cognate interactions of the memory CD4^+^ T lymphocytes with antigen‐presenting cells. The reactivated memory T lymphocytes start to proliferate vigorously for several days. Then the clusters dissociate. The amplified memory T lymphocytes relocate to memory niches and return to proliferative and functional rest. Such immune clusters^131^, ^152^ or immune niches^153^ had been observed before, but their context had remained obscure. It seems that they serve mainly to amplify the antigen‐specific memory T lymphocytes. Although B cells are found in the clusters, they are not specific for the antigen memorized, nor are they differentiating into germinal center‐like cells.^59^, ^151^ Accordingly, the reactivated memory T lymphocytes also do not differentiate into follicular helper T cells.^151^ Whether or not the reactivated memory T cells of the bone marrow also emigrated into the secondary lymphoid organs and contributed to the secondary immune responses there, is not clear.

Since most cells of the body express MHC class I, CD8^+^ memory T lymphocytes are contacting MHC class I‐expressing cells in the bone marrow, also in the memory phase of an immune response. Their reactivation and their contribution to secondary immune reactions have not been analyzed so far.

## 8 “TISSUE‐RESIDENT” VS BONE MARROW‐RESIDENT MEMORY T LYMPHOCYTES

It is interesting to note that in the recent hype about “tissue‐resident” memory T lymphocytes, bone marrow‐resident memory T lymphocytes were largely disregarded, quasi the “Cinderella” of memory lymphocytes.^154^, ^155^, ^156^, ^157^, ^158^, ^159^

In a restricted sense, the term “tissue‐resident” has been used by others for antigen‐experienced T lymphocytes, in particular CD8^+^ lymphocytes, of the skin, gut, lung, and other peripheral tissues, mostly barrier tissues. In absolute numbers, the human skin harbors almost twice as many CD45RO^+^ T cells as the peripheral blood.^160^ Human bone marrow contains 3‐4 times as many CD45RO^+^ T cells as peripheral blood.^33^ In peripheral tissues and in the bone marrow, the numbers of experienced T cells recognizing a particular antigen remain high over time, while numbers of their circulating counterparts drop. Populations of “tissue‐resident”, antigen‐experienced T cells of lung, gut, brain, thymus, and skin express CD103 (αE integrin) and CD69.^154^, ^155^ CD103 mediates binding to E‐cadherin, which is expressed on epithelial cells.^161^ Mice lacking CD103 show defects in establishing antigen‐experienced “tissue‐resident” T cells of peripheral tissues.^162^ For the establishment of bone marrow‐resident memory CD4 lymphocytes, we have shown that CD69 is essential.^137^ “Tissue‐resident” memory T cells have downregulated S1PR1, like CD69^+^ bone marrow memory T cells.^33^ “Tissue‐resident” memory T cells, despite expressing CD69, rest in terms of proliferation and expression of effector molecules.^160^, ^163^ “Tissue‐resident” memory CD8^+^ T lymphocytes have a typical gene expression signature, and express the transcription factor Hobit.^134^, ^159^ The signature genes of “tissue‐resident” memory T cells are also expressed by bone marrow‐resident memory T cells.^151^ That “tissue‐resident” memory T cells are indeed resident, ie, do not circulate, has been suggested by parabiosis experiments.^164^ For human “tissue‐resident” memory T cells of the skin, it has been argued that CCR7‐negative cells are residents of the skin, since they are refractory towards treatment with anti‐CD52 (Alamtuzumab), depleting circulating memory T cells, including CCR7‐expressing memory T cells of the skin.^165^ Aside from this notion, the lifestyle of “tissue‐resident” T cells is not so clear. Antigen is needed for the differentiation of T cells into “tissue‐resident” T cells.^157^, ^166^ They seem to play a prominent role in controlling persistent, latent infections,^167^ but apparently can also be maintained in the absence of antigen.^162^ “Tissue‐resident” memory T cells have been postulated to survive in dedicated niches providing them with distinct survival signals, such as CD103/E‐Cadherin^166^ or CD49a/Collagen type I and IV.^168^ This would be quite similar to the concept of memory T lymphocytes resident and resting in bone marrow, as discussed above. The concept of resident and resting memory T cells, however, is hard to combine with observations suggesting that tissue‐resident memory T cells constantly migrate through the tissue, scanning it for cells presenting them their antigen.^169^

Taken together, memory T cells residing in peripheral barrier tissues are considered to provide frontline immunity against pathogens invading those tissues. Their lifestyle seems to be similar to that of memory T cells residing in the bone marrow. Bone marrow memory T cells apparently provide immunity against systemic challenges. What is less clear is the role of the circulating memory T lymphocytes. Are they providing a “short‐term” memory, which could last even for years, but declines over time and eventually disappears? Is this the memory maintained in secondary lymphoid organs themselves, like in the spleen and lymph nodes, and leaving them from time to time to circulate through blood and lymph?

## 9 MEMORY B LYMPHOCYTES OF THE BONE MARROW

Does bone marrow also host memory B lymphocytes, and if so, are these circulating or resident? Again, at first glance, the B lymphocyte memory may be considered as consisting of long‐lived, circulating cells, nesting in secondary lymphoid organs.^170^, ^171^, ^172^ For influenza‐specific memory B lymphocytes, the preferential localization in the affected barrier tissue, the lung, has been described.^173^, ^174^ The lifestyle of memory B lymphocytes in secondary lymphoid organs is still enigmatic. By switching the specificity of their surface antibodies through Cre‐mediated recombination, the group of Rajewsky has elegantly demonstrated that the persistence of memory B lymphocytes is independent of persisting antigen.^175^ Non‐cognate signals essential for the maintenance of memory B lymphocytes have not yet been identified.

Memory B lymphocytes display a considerable diversity, as reviewed by the groups of Reynaud and Weill^176^, ^177^ and Küppers,^178^ pointing also to differences between mice and humans. Memory B lymphocytes expressing switched isotypes of antibodies and IgM‐expressing memory B lymphocytes have been identified. Memory B lymphocytes undergoing further affinity maturation in germinal center reactions, and memory B lymphocytes independent of germinal centers, have been described.^179^, ^180^

Memory B lymphocytes of the bone marrow have not been analyzed extensively.^181^, ^182^ Paramithiotis and Cooper showed a preferential localization of IgM and IgD expressing CD24^low^CD10^−^ B lymphocytes with somatically mutated antibody genes indicative of antigen experience in the bone marrow.^182^ Giesecke and colleagues compared numbers and phenotypes of memory B lymphocytes from various human organs, including the bone marrow. For switched memory B cells, which were present in blood, spleen, tonsils, and bone marrow in about equal numbers, they did not find phenotypic differences. Tetanus‐specific memory B cells were present in all organs analyzed. They concluded that switched memory B lymphocytes of all organs were part of one, circulating population of memory B lymphocytes.^181^ However, a comprehensive repertoire analysis of memory B lymphocytes of various organs, with the aim to identify resident memory lymphocytes, has not been performed yet. In light of the present recognition of resident memory T lymphocytes of the bone marrow with an exclusive repertoire for systemic pathogens encountered long ago, it would not be too surprising if a population of complementary resident memory B lymphocytes will be identified in the future.

## 10 PROTECTION OF IMMUNOLOGICAL MEMORIES BY BONE

The analysis of memory plasma cells of bone marrow has generated a novel understanding of how immunological memory is organized by mesenchymal stromal cells, counting memory cells and defining their populations, by direct individual cell contacts. Persistence of the memory cell is conditional on signals from these niches, and not on intrinsic half‐lives. Evidence is accumulating that also memory T lymphocytes are maintained in this way, at least those residing in the bone marrow, probably also those residing in barrier tissues. We are still lacking detailed information on the molecular codes of memory niches and their diversity, a challenge for future research. We also need to obtain an understanding of the lifestyle of circulating memory T lymphocytes and memory B lymphocytes. Beyond that, the role of the bone marrow in the maintenance of experienced innate lymphocytes awaits analysis, a topic beyond the scope of the present review.^183^, ^184^

Recruitment of memory lymphocyte precursors to memory niches, their reactivation and their role in secondary immune reactions are further challenges in this renaissance of research on immunological memory. It is not just an academic exercise. The promise is to develop effective vaccines against pathogens for which we do not yet have such vaccines, and to develop novel therapies against immune‐mediated diseases which are driven by pathogenic memory plasma cells and memory lymphocytes, like chronic inflammatory and allergic diseases. Already now, lifestyle‐based targeting of memory plasma cells secreting pathogenic autoantibodies is starting to fulfill these promises (reviewed in Ref. 24, 25).^185^, ^186^

## CONFLICT OF INTEREST

The authors declare no conflict of interest.
